# Natural Architectures for Tissue Engineering and Regenerative Medicine

**DOI:** 10.3390/jfb11030047

**Published:** 2020-07-07

**Authors:** Floris Honig, Steven Vermeulen, Amir A. Zadpoor, Jan de Boer, Lidy E. Fratila-Apachitei

**Affiliations:** 1 Laboratory for Cell Biology-Inspired Tissue Engineering, MERLN Institute, University of Maastricht, 6229 ET Maastricht, The Netherlands; florishonig@gmail.com (F.H.); s.vermeulen@maastrichtuniversity.nl (S.V.); 2 BioInterface Science Group, Department of Biomedical Engineering, Eindhoven University of Technology, 5600 MB Eindhoven, The Netherlands; j.d.boer@tue.nl; 3 Biomaterials and Tissue Biomechanics Section, Department of Biomechanical Engineering, Faculty of Mechanical, Maritime, and Materials Engineering, Delft University of Technology, 2628 CD Delft, The Netherlands; A.A.Zadpoor@tudelft.nl

**Keywords:** natural and nature-inspired surfaces, surface-cell interactions, biomimicry, (bio) materials, tissue engineering, regenerative medicine

## Abstract

The ability to control the interactions between functional biomaterials and biological systems is of great importance for tissue engineering and regenerative medicine. However, the underlying mechanisms defining the interplay between biomaterial properties and the human body are complex. Therefore, a key challenge is to design biomaterials that mimic the in vivo microenvironment. Over millions of years, nature has produced a wide variety of biological materials optimised for distinct functions, ranging from the extracellular matrix (ECM) for structural and biochemical support of cells to the holy lotus with special wettability for self-cleaning effects. Many of these systems found in biology possess unique surface properties recognised to regulate cell behaviour. Integration of such natural surface properties in biomaterials can bring about novel cell responses in vitro and provide greater insights into the processes occurring at the cell-biomaterial interface. Using natural surfaces as templates for bioinspired design can stimulate progress in the field of regenerative medicine, tissue engineering and biomaterials science. This literature review aims to combine the state-of-the-art knowledge in natural and nature-inspired surfaces, with an emphasis on material properties known to affect cell behaviour.

## 1. Introduction

Over the course of evolution, nature developed various biological materials that are optimised to serve a wide variety of functions. For example, spiders can produce different types of silk with varying mechanical properties to capture preys [1], honey bees build highly self-organised patterned honeycombs for efficient habitation [2], and shells provide the primary means of protection for the soft bodies of the animals they house [1]. Moreover, animals consist of different specialised tissues (e.g., tendons, bones, and skin) and sponges do not need nervous, digestive, and circulatory systems because of the pores and channels in their bodies [1]. In the plant kingdom, superhydrophobic waxes allow self-cleaning, with particle reduction and antimicrobial effects [3]. Biomimicry or bioinspiration is the development of novel technologies through transferring function from these biological systems and can solve many complex problems faced by humanity across numerous disciplines [4]. For instance, naturally occurring proteins in animals and plants inspired scientists to promote tissue healing in humans using nanofibre scaffolds [5]. In addition, honeycomb structures allowed engineers to create materials with a high strength-to-weight ratio, which is useful in biomedicine for the design of 3D porous structures for tissue engineering [6]. Furthermore, soft materials in animals such as the octopus stimulated the development of a new type of adaptive robotics based on their highly flexible and deformable properties [7,8].

In the field of regenerative medicine and tissue engineering, an important area of interest is the development of functional biomaterials for directing cell fate [9,10,11,12]. In vitro tissue construction can be hindered by a loss of phenotypic characteristics of the primary cells culture [13,14]. Additionally, when primary cells are unavailable, differentiating stem cells towards specialised cell types through material cues offer an interesting opportunity for regenerative therapies [15]. In vivo, controlling immune cell behaviour is necessary to avoid foreign body reactions, which eventually can lead to decreased performance of implanted biomaterials though material encapsulation [16]. In general, cells respond to different physical and biochemical cues in the ECM, such as structure, stiffness, adhesiveness, degradability, biochemical composition, and ligand adsorption [15,17,18,19,20,21]. Hence, modulating the inherent properties of biomaterials plays an important role in controlling cell behaviour. However, the mechanisms underlying the interplay between material properties and cell phenotype are complex. This makes it difficult to identify optimal surface characteristics for both in vitro and in vivo applications.

In the age of increased antibiotic resistance due to overuse and misuse of antibiotics [22], the need for alternative methods to ward off bacterial contamination on medical implants is growing. These bacterial infections have serious adverse effects on the efficacy of biomaterials in various clinical settings [23,24]. Treatments of such infections are challenging because of the different resistance mechanisms existing in bacteria [25]. In addition, antibiotic resistance causes clinical and societal problems associated with high healthcare costs [26,27]. Therefore, for tissue engineering applications, antimicrobial biomaterials gain specific interest in mitigating microbial surface colonisation besides the focus on controlling cell behaviour. It is known that biofilm formation can be prevented via chemical or physical modifications. Chemical approaches incorporate biocidal materials, such as nanoparticles [28,29,30] and polymers [31,32], to resist microbial colonisation. Physical methods on the other hand alter surface topographical parameters, including aspect ratio [33], roughness [34,35] and geometry [36], to induce spatial cues that combat biofilm formation. However, despite the advancement in the design of antimicrobial biomaterials as mentioned above, a real consensus on the ideal surface criterion to avert bacterial infections has not been reached.

To tackle these problems, artificial high-throughput systems were developed for identifying surface properties with a most optimal outcome [37,38,39,40]. This approach can be applied both on a structural and chemical level. In addition, high-throughput platforms exist to screen for desirable properties in the field of material science [41,42,43]. Although high-throughput platforms offer an unbiased method for discovering optimal material properties, they have their limitations since only a restricted part of the material design space is covered. For instance, both the surface topographical design space and chemical diversity is immense. For surface architectures, patterns can be constructed on both nano- and microlevel dimensions, with different heights, densities, and shapes in either an ordered or disordered manner. For polymers, a large diversity exists in combining different monomer blends together. This is illustrated for polyurethane, a polymer commonly used for clinical applications, of which already hundreds of varieties exist that can evoke different cell responses [44]. Thus, a key challenge remains in identifying suitable material properties for generating a specific biological response.

In an alternative approach, natural surface materials that through millions of years of evolution carry specialised properties, can inspire material scientists for designing novel materials for tissue engineering, regenerative medicine, and biomaterials applications. In this review, we provide the reader with an overview of natural material properties that can be harnessed for these applications with a special emphasis on surface topography.

## 2. Natural Surfaces

**Figure 1:**
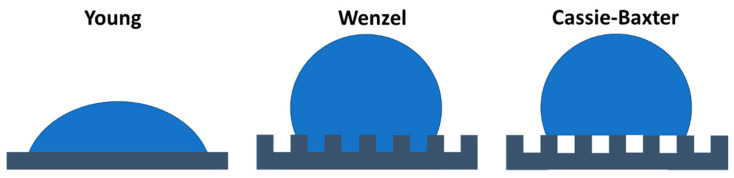
Different models describing wetting behaviour on solid substrates depending on surface structure. Compared to an ideal solid surface (Young, **left**), surface roughness and topography affects wetting behaviour either by increasing the contact area of the solid-liquid interface (Wenzel, **middle**) or by introduction of a liquid–vapour interface because of trapped are underneath the water (Cassie-Baxter, **right**).

Natural surfaces have received a tremendous amount of attention because of their special wettability, including anisotropic wetting and superhydrophobicity [45,46,47]. In general, wettability is determined via measurements of contact angles (CA) when liquid interacts with a solid surface according to Young’s model (Figure 1, left). On natural surfaces, this wetting behaviour is highly affected by the inherent surface roughness and topography, as already described by the Wenzel [48] and Cassie-Baxter [49] models in the mid-1900s. In the Wenzel model, the water protrudes into the gaps of the rough surface [48] (Figure 1, middle), whereas in the Cassie-Baxter model air is trapped in the valleys underneath the water [49] (Figure 1, right). For theoretical background on these wettability models some excellent works are available [50,51,52,53]. In the next section, several examples of natural surfaces with unique surface properties are given, which are often linked to wettability.

### 2.1. Self-Cleaning, Superhydrophobic, and Ultrahigh Pinning Properties in Plants

**Figure 2:**
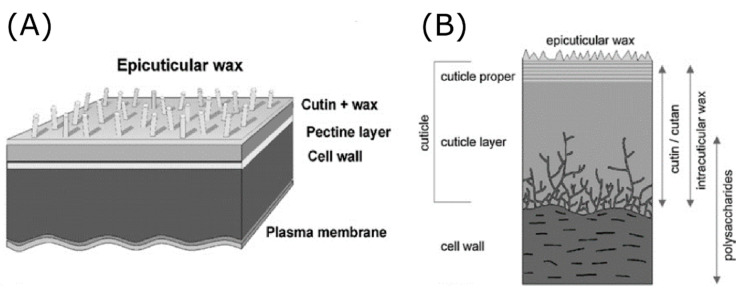
Stratification of the outer layer of the plant surface. (**A**) The cuticle is connected to the cell wall through a pectin layer. The epicuticular waxes on the cuticle establish the structural features of the plant surface. (**B**) The cuticle is composed of cutin and cuticular waxes, which vary in chemical and structural composition among different plant species. Reproduced from Ref. [59] with permission from The Royal Society of Chemistry.

To date, plant biodiversity is approximated at 270,000 different species worldwide [3]. Adaptation to environmental conditions for over millions of years has resulted in a large variety of multifunctional biological surface structures among these plants [3]. For example, a study covering 200 water repellent plants identified diverse surface structures depending on their origin [54]. Plants have been a source of inspiration for biomimetics for several decades. Well-known functional aspects include the reduction of particle adhesion, self-cleaning properties and anti-pollution effects, based on the physico-chemical surface properties of plants [55]. Such properties are created by the chemistry and structure of the most outer layer of the plant surface, which is composed of the cuticle (Figure 2). This part varies in roughness, topography, hierarchical structure, and chemistry among distinct plant species [56]. The cuticle, better known as the protective film covering the epidermis of plants, consists of two main components: the cutin and the cuticular waxes. Cutin is a polyester of hydroxylated fatty acids (C_16_ and C_18_) and glycerol [55], whereas the cuticular wax is a mixture of diverse hydrocarbon chains and rings [55]. Generally, epidermal cells cause variation in structure at the microscale, whereas cuticle morphologies differ at the nanoscale. The most common nanowaxes have three-dimensional structures described as crusts, granules, plates, platelets, filaments, rods, and hollow tubules with sizes ranging from 0.2–100 µm [56]. Examples of epidermal cell morphologies include: hemispherical, cupola, cone-shaped, papilla, and hair [3]. As said before, the structural basis formed by these components establishes important functional effects in plants, including superhydrophobicity. This physical property of the leaf cuticle was first described in 1944 [57]. It was observed that changes in the surfaces properties of the cuticle were related to alterations in the closeness of packing of hydrophilic and hydrophobic units [57]. In nature, superhydrophobic properties play an important role in establishing self-cleaning and anti-pollution effects [58]. Superhydrophobicity is characterised by an apparent water CA larger than 150°. Here, three recognised superhydrophobic plants are briefly highlighted because of their special surface properties and wettability.

**Figure 3:**
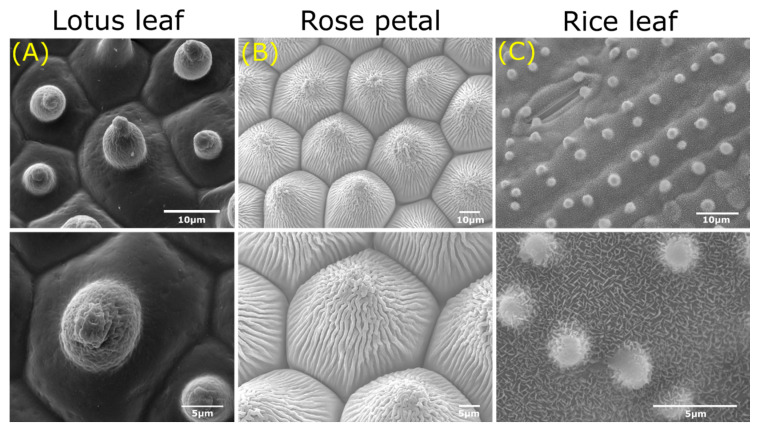
Overview of superhydrophobic plants found in nature displaying unique hierarchical structures. SEM images depicting the distinct micro- (top) and nanostructures (bottom); papillae and tubules of the sacred lotus (*Nelumbo nucifera*) (**A**), papillae, and cuticular folds of the red rose (*Rosea rehd)* (**B**), ridges and papillae of the rice plant (*Oryza sativa*) (**C**). Scale bars: top 10 μm, bottom 5 μm. Images adapted from: Vermeulen et al. [63].

First of all, one of the most famous plants with superhydrophobic leaves is the sacred lotus (*Nelumbo nucifera*) [60], which shows high water repellence and self-cleaning effects. The removal of dust particles by water droplets that roll over the surface of the lotus leaf have led to the concept of the “lotus-effect” [60]. Multiple studies show that the superhydrophobicity of the lotus plant is a consequence of the micro- and nanostructure present on the surface of the upper epidermis [61,62]. The hierarchical structure consists of papillae at the microscale, while the nanoscale is characterised by epicuticular wax tubules (h: 0.1–3 µm, w: 80–120 nm) [62] (Figure 3A).

The shape of these hollow wax tubules found on the lotus plant surface is dependent on its chemical composition, which are mainly secondary alcohols such as nonacosan-diols [64]. The structural duality traps a thin layer of air between the papilla, resulting in high water repellence, according to the Cassie-Baxter model [49]. Of interest, lotus leaves show lower wettability compared to other plant species for low surface tension liquids [65]. The surface roughness on different length scales inherent to papillose plant surfaces is key to this liquid repellence [65]. Altogether, the sacred lotus leaf shows self-cleaning effects due to its superhydrophobic properties caused by distinct hierarchical surface structures.

In contrast to lotus leaves, petals of red roses (*Rosea rehd*) possess, next to superhydrophobic properties, ultrahigh water pinning forces [66,67,68]. This “petal effect” allows the immobilisation of water droplets on rose petals, even when turned upside down [68]. Just like the lotus leaf, the properties of the rose petal are the consequence of a dual surface structure. This hierarchical structure consists of micropapillae *(h*: 7 µm, *d*: 16 µm) that exhibit cuticular folds (*w:* 730 nm) at the nanoscale (Figure 3B) [68]. The high adhesive force is due to the large contact area between the water and the rose petal’s surface as the water droplet protrudes entirely into the nanofolds (Wenzel model [48]). Moreover, the micropapillae control the degree of liquid–solid adhesion [66]. Summed up, the rose petal combines papillae and nanogrooves to create a superhydrophobic surface with high pinning forces.

The rice leaf (*Oryza sativa*) is recognised due to its anisotropic wettability and superhydrophobic properties [69,70,71]. Like the lotus leaf, water droplets roll off the surface of the rice leaf, ensuing self-cleaning and draining processes [70]. Of interest, rice leaves can only shed water droplets along the longitudinal direction of the leaf. Again, this behaviour originates from the multiscale surface roughness and chemical hydrophobicity. The upper side of the rice leaf is characterised by vascular bundles forming parallel ridges (*h*: 125–150 µm *w*: 150–175 µm) on which several micropapillae (*h*: 2–4 µm, *d*: 2–4 µm) are displayed covered by nanowaxes (Figure 3C) [72]. The platelet shape of the wax is associated with the aldehyde composition of the wax [73]. Besides, the leaf contains sub-cuticular features composed of silicon oxide which have favourable effects on the mechanical and physiological properties of the rice plant [3]. The anisotropic rolling behaviour is highly dependent on the roughness aspect ratio and directionality of the micropapillae [74]. Overall, the anisotropic rolling properties result from the hierarchical structure and directional microstructures of the rice leaf.

In short, the combination of surface roughness at micrometer dimensions together with varying properties of the cuticle components at the nanometer range are the basis of the surface structure of plants and bring about their unique properties. The many kind of cuticular waxes give rise to distinct types of wetting behaviour as described for the sacred lotus, red rose, and rice plant. These three plants function as examples to highlight the different types of superhydrophobicity found in plants.

Such superhydrophobic properties can be used for antimicrobial applications [75,76,77,78,79]. At the moment, the hierarchical structure of the sacred lotus is utilised in the design of antifouling surfaces with potential applications in industrial, marine and medical fields [75,76,77]. By mimicking the previously described ‘lotus-effect’, researcher are able to prevent the adhesion of bacteria and algae to these surfaces [75,76]. Of interest, titanium surfaces with copied lotus structure, which is a commonly used material for orthopaedic implants [80], also showed antifouling effects [77]. Similarly, the nanostructure of the taro (*Colocasia esculenta)* prevents fouling of bacteria and colloids [78]. Likewise, another study reported the ability of sixteen reproduced plant surfaces to affect the spatial distribution of *Pseudomonas aeruginosa* attachment [63]. Superhydrophobic characteristics of plants are also exploited for self-cleaning and drag-reducing effects [81,82]. For example, Xiang et al. [81] fabricated a biomimetic *Salvinia molesta* surface using a 3D printing approach, which imitates the floating fern’s superrepelent capability. Similarly, the rice leaf anisotropic structure has been implemented for such properties [82]. Altogether, bioinspired design using micro- and nanostructures present on plant surfaces are useful for antimicrobial effects.

### 2.2. Self-Cleaning, Antifouling, and Special Wettability in Insects

According to recent estimates, the amount of insect species is estimated at 5.5 million [83]. Interestingly, the largest study on surface structures found on insect wings only covers 97 species [84]. Among those, a great diversity in surface structures and special abilities can be identified. For example, nanopillars on cicada wings limit bacterial contamination through self-cleaning [85], scales of butterflies induce structural colonisation [86], termites can undergo a colonisation flight due to micrasters on their wings [87], water striders perform hydrodynamic propulsion facilitated by needle-like structures [88], and beetles capture water from fog using arrays of bumps on their elytra [89] because of structural adaptations made over time to cope with environmental stresses. Insect wings have especially received attention due to their highly sophisticated structures [84]. Similar to plants, the surfaces of insects consist of cuticular layers with different surface topography and chemistry [73]. However, contrary to plants, the cuticle is mainly composed of chitin and protein [73]. At the end of the 20th century, Wagner et al. [84] examined wings of 96 insect species in order to find a relationship between the wing surface structures and their wettability and contaminability. Several morphologies were identified, ranging from hair-like structures to plate-like scales and tooth sculptures. More recently, the morphologies of wax crystals on the insect wing surfaces are categorised as setae, denticles, and fractals [73].

**Figure 4:**
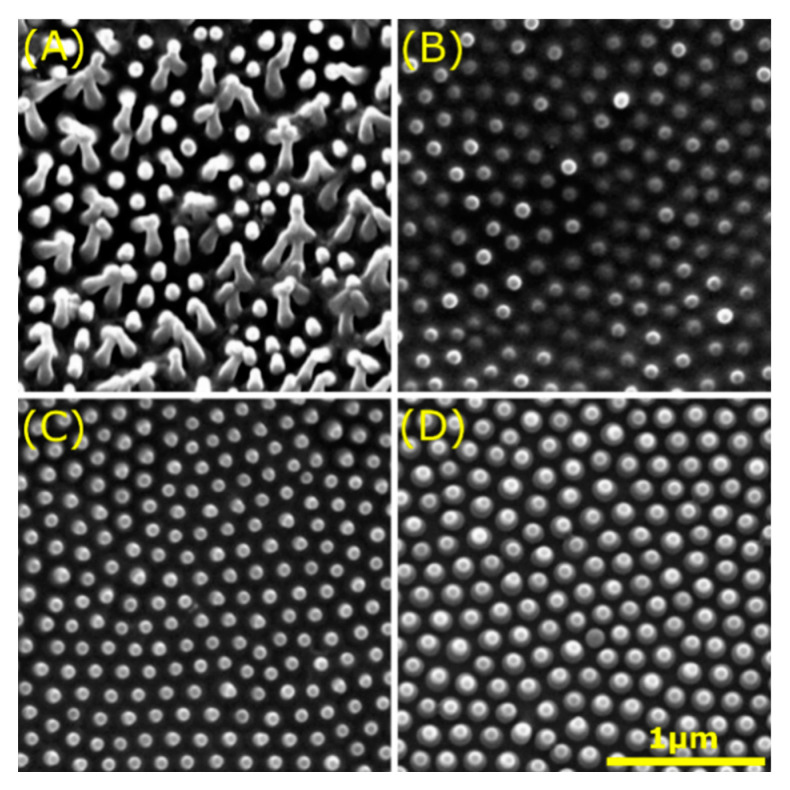
Collage of four types of protrusions found on cicada wings. SEM analysis reveals the distinct surface topographies on the wings of the *Chremistica maculate* (**A**), *Mogannia conica* (**B**), *Meimuna microdon* (**C**), and *Terpnosia jinpingensis* (**D**). Scale bar: bottom right. Images adapted with permission from: *J. Exp. Biol.,* M. Sun et al. [90].

Cicadas gain specific attention due to the irregular nanostructures present on their wings. For example, Sun et al. [90] investigated the wettability of 15 species of cicada, identifying both hydrophilic and hydrophobic wings depending on the size and arrangement of the protrusions (*d*: 82–446 nm) (Figure 4). In general, structures with greater height and diameter but smaller spacing exhibited hydrophobic properties. Hydrophilic wings are a result of a more disordered type of surface patterning, giving a larger solid-liquid interface. Interestingly, some cicada wings displayed CA values (137–146°) associated with superhydrophobicity [90], which are related to self-cleaning [51] and antifouling [91] mechanisms. However, other studies show that cicada limit bacterial attachment directly due to the physical surface structure present on their wings, even independent of chemistry [85,92]. Similar as with inducing variable hydrophobicity levels, the unique scale of the topography, associated with small pitch (165–251 nm) and spacing (9–44 nm), prevented bacterial cell adhesion [92].

**Figure 5:**
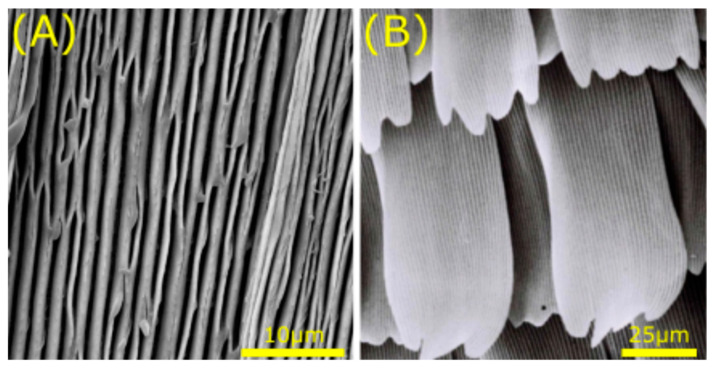
Structural representation of the butterfly wing. SEM images show the microgrooves on the scale structure of the *Morpho anaxibia* (**A**) and the rooftop arrangement found on the *Pontia daplidice* (**B**). Scale bars: bottom right. Image adapted by permission from Springer Nature Customer Service Centre GmbH: Springer Nature, J. Bionic Eng, Anisotropism of the Non-Smooth Surface of Butterfly Wing, G. Sun et al., © (2009) [96].

Butterflies, another species of insects, show similar anisotropic wetting behaviour as previously described for the rice leaf [79,93]. Superhydrophobic properties together with low adhesion are provided by microgrooves on the scale structures of butterfly wings (Figure 5A). The hierarchical structures shows directional adhesion, making a water droplet roll off in the radial outward direction and pin in the radial inward direction [94]. The wing is composed of scales arranged like rooftops as shown in Figure 5B, forcing anisotropic wettability. Furthermore, the multilayers and scales in butterfly wings also cause multilayer interference and diffraction, resulting in a broad spectrum of structural colours. Prum et al. [86] examined the structural colours of 11 butterfly species, identifying 13 distinct wavelengths. For all species, the scales are aligned as shingles on the upper surface of the wings. Investigation of the anatomy and nanostructure of the wings revealed a great diversity in shape of the scales, which affect the refractive index of the tissue. Lastly, Goodwyn et al. [95] found a link between wing colour and wettability. Namely, translucency and hydrophobicity are both affected by scale cover [95]. While reduced scale cover in wings increases translucency, hydrophobicity performance is decreased. Similar as with plant surfaces, this highlights the importance of the spacing and size of nanostructures on wettability.

**Figure 6:**
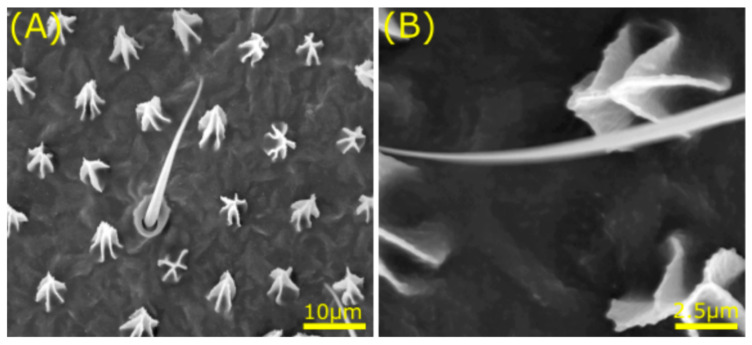
Micro- and nanostructures on the termite wing. SEM analysis reveals contrasting micro- (**A**) and nanostructures (**B**) that induce special wettability on the termite wing (*Nasutitermes sp.*), including hairs and micrasters. Scale bars: bottom right. Image adapted from: © (2011) Watson et al. [87].

Another insect with contrasting micro- and nanostructures on its wings is the termite (Figure 6) [87,97]. Termites continuously deal with rain periods and fly from their nests during such occasions of rainfall. Because of the changing environmental conditions and their lack of ability to fly for longer periods, termites have evolved special wettability on their wings. Hydrophobic termites are characterised by hairs and smaller structures on their wings termed micrasters, composed of 5–7 arms of approximately 100 nm which highly influence wetting behaviour [87]. Microdroplets on these types of wings form a Cassie-Baxter type of interaction. Moreover, higher structures allow higher hydrophobicity of the wing surfaces, which was also observed in cicada wings described earlier. Contrary, wings of hydrophilic termites consist of folds and ridges with topographies arranged in a hexagonal fashion next to curved perturbances spaced 700–1200 nm apart and 150–250 nm in height [87]. In short, surface topography guides wetting behaviour, where micrasters support hydrophobicity and hexagonal structures lead to hydrophilicity.

**Figure 7:**
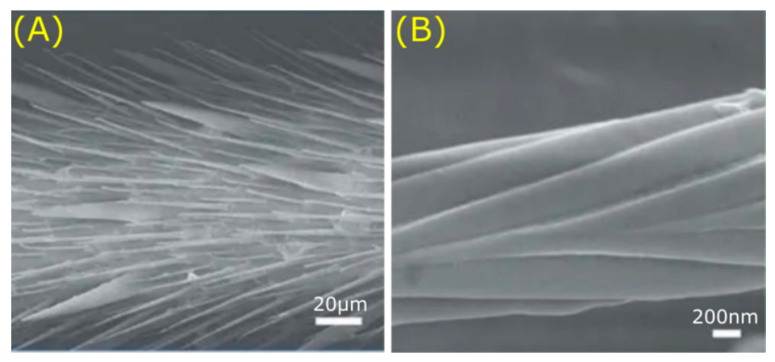
Hierarchical structure on the legs of the water strider. Various microsetae (**A**) with fine nanogrooves (**B**) lead to non-wetting legs. Scale bars: bottom right. Image adapted by permission from Springer Nature Customer Service Centre GmbH: Springer Nature, Nature, Water-repellent legs of water striders, Gao et al., © (2004) [100].

Water striders have the ability to walk on water, which is made possible by thousands of needle-like structures known as setae on their legs (Figure 7) [98,99]. These setae are oriented at an angle of inclination of approximately 20° with respect to the leg surface, with a length of 50 µm and a diameter of 3 µm [98]. The roughness and hierarchical structure of the leg surface results in superhydrophobic properties that can induce hydrodynamic propulsion to move on the water [88]. These properties enable the water strider to survive on water even during heavy rainfall.

Some beetles (e.g., *Stenocara*) situated in the Namib Desert use fog as an alternative water source due to low rainfall [89,101]. During the morning fog, large water droplets form on the surface of the beetle, which is composed of alternating hydrophobic and hydrophilic regions [89]. The mechanism works by producing droplets on the hydrophilic regions of the elytra, which increase in size and roll down to the mouth of the beetle. The microstructure consists of hemispheres (*d*: 10 µm) arranged in hexagonal fashion, which shows some resemblance to the previously described structure of the lotus leaf [89]. The hydrophobic regions are covered by wax, whereas on the hydrophilic regions wax is absent [101]. Next to the presence of wax, rougher elytra surfaces characterised by irregularities caused by cracks, hairs and pores also influences the wettability, showing stronger hydrophobicity [102]. For optimal fog collection, the beetle can undergo a fog-basking posture oriented head down at a 23° angle [101]. Altogether, the beetle efficiently collects water from fog through a system guided by structures found on their elytra.

Overall, insects show a great variety in surface roughness, chemistry, and topographies. Distinct morphologies at the micro- and nanoscale give insects self-cleaning, antifouling, special wettability, fog-collecting, and walk on water abilities as mentioned in the above described insects. These surface characteristics of insects can be implemented in material design for different applications [82,103,104,105,106]. For instance, Zhai and colleagues [106] successfully fabricated a surface that mimics the water collecting behaviour of the beetles in the Namib desert by copying the structure seen on their elytra. Possible applications of such a surface include coatings for controlled drug release and microfluidic devices. Surface structures found on insect wings can also be used for the design of antifouling surfaces [63,82,103,104,105]. For example, nanopatterns, ranging from hexagonal arrays of nanopillars [104] to diamond nanocones [105], inspired by the cuticles found on insect wings, display such properties. Likewise, replicated superhydrophobic dragonfly and cicada wings show resistance to biofouling [103]. Similarly, bactericidal activity of black silicon is based on high aspect ratio nanoprotrusions also seen in the dragonfly [107]. A comparable effect on the attachment of *Pseudomonas aeruginosa* was observed on ten replicated insect surfaces [63]. Lastly, the scale structure of the butterfly wing has been used for low-drag and self-cleaning purposes [82]. These examples demonstrate that the unique surface characteristics of insect wings have applications in several fields.

### 2.3. Special Wettability, Low Drag, and Structural Absorption in Vertebrates

**Figure 8:**
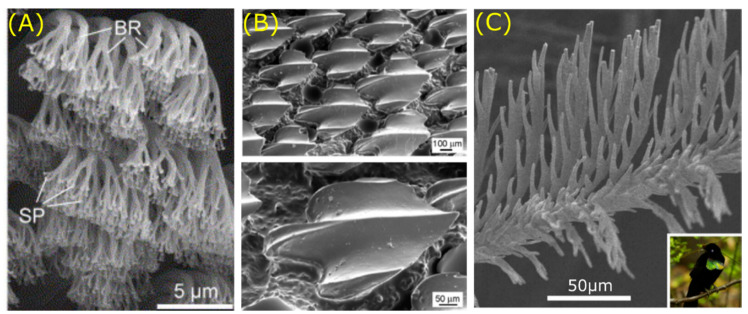
SEM display of different microstructures found in animals. (**A**) The hierarchical structures of the gecko (*Gekko gecko*) consists of setae made up of branches (BR) and spatulae (SP), enabling it to walk on vertical surfaces. (**B**) Riblets found on the scales of sharkskin (*Squalus acanthias*) reduce friction drag in the direction of the flow. (**C**) Modified barbule arrays on the feathers of a bird of paradise (*Parotia wahnesi*) cause structural absorption resulting in a super black appearance. Scale bars: bottom right. Images adapted with permission from: (**A**) Mech. Mater., 37, Gao et al. [110], Mechanics of hierarchical adhesion structures of geckos, 275–285, © (2005) Elsevier. (**B**) Jung et al. [113], Biomimetic structures for fluid drag reduction in laminar and turbulent flows. J. Phys. Condens. Matter, 22, 1–9, © (2010) IOP Publishing. (**C**) McCoy et al. [114] under the Creative Commons Attribution 4.0 International License.

The gecko has not only generated interest because of its remarkable solid-solid adhesion to vertical surfaces [108], but also due to its liquid–solid superhydrophobicity and high adhesive forces towards water droplets [109]. The ability of the gecko to walk on vertical surfaces is facilitated by a system consisting of setae (30–130 µm) covered by spatulae (200–500 nm) (Figure 8A) [110]. The high density of the spatulae enables high adhesion strength, while the setae provide initial attachment force. This hierarchical adhesive structure is able to adapt to different substrates depending on their surface roughness [108]. As the adhesive system of the gecko is facilitated by Van der Waals interactions, increased surface density results in greater adhesive forces [111]. The spatulae must be able to contact the substrate to achieve maximum adhesion strengths. Therefore, greater surface roughness values of the spatulae allow greater contact area that enhances adhesion [108]. In addition, the asymmetric nature of the setae structure allows quick attachment and detachment at necessary angles to prevent contact flaws [110]. Superhydrophobicity of the gecko feet can is attributed to the multiscale structure formed by the setae and spatulae [109]. The high adhesive forces towards water are a result of heterogeneous morphology and orientation of the structures as explained by Liu et al. [109]. In short, the high-density spatulae create a high adhesive force towards water. Next to the feet of the gecko, its skin also displays superhydrophobic properties due to microstructures featuring spinules (*l:* 4 µm), thereby controlling liquid, solid, and biological contacts [112]. The gecko thus evolved surface structures at different scale levels for specialised functions, either to achieve robust and reversible attachment or for self-cleaning purposes.

In recent years, sharkskin gained attention due to its antifouling and drag reducing properties [113,115]. The riblets on a sharkskin are oriented in the flow direction in order to reduce friction drag, as summarised by Dean and colleagues [115]. The sharkskin surface structures are directional through riblets that are aligned along the swimming direction. The riblets, also known as dermal denticles, are organised in small ridges with longitudinal grooves (Figure 8B). The height of these riblets ranges from 200–500 µm, with a spacing varying between 100–300 µm [113]. Moreover, the riblet structure also protects sharks against biofouling [116]. This is due to the low drag properties of the sharkskin and the spacing and structure of its riblets. Lower drag results in faster water movement, which reduces the settlement time for microorganisms. In addition, the riblet microstructure deters microorganisms, as the sharkskin’s groove width and depth is not preferred [116]. This behaviour has been confirmed on biomimetic sharkskin surfaces [117]. Altogether, the microstructures on the sharkskin reduce friction drag, exhibit hydrophobicity and attribute to antifouling effects.

Remarkable material properties can also be found in the feather of birds [118,119]. For example, water repellent properties of diving birds were identified by Gremillet and colleagues almost a decade ago [118]. The birds maintain a thin layer of air in their plumage due to two distinct zones. The inner part shows a regular feather structure, whereas the outer part possesses an irregular structure. This duality provides the birds with a waterproof inner section and a wettable outer section [118]. Similar to diving birds, the outer feathers of pigeons also show special wetting behaviour [119]. The barbs and barbules of the pennae create a Cassie-Baxter type of wetting regime for small water droplets. The multiscale surface forces rain drops to roll off the feather, making it waterproof. Besides special wettability, feather structures can also influence the appearance of birds. Of special interest, McCoy et al. [114] showed that feathers of five Birds of Paradise structurally absorb light to produce a super black appearance. In comparison, the birds show the same extremely low directional reflectance as seen in man-made super black materials based on carbon [120]. Multiple scattering of light caused by the tilted barbule microstructures in feathers results in more structural absorption than in other birds (Figure 8C). These structures have evolved over the years, because the super black plumage enhances the bird’s courtship display. Overall, the feathers of birds possess distinct microstructures that influence both wetting behaviour and appearance, as earlier observed for wings of butterflies.

The above described properties found in vertebrates can be useful for many applications. For example, the adhesion of gecko pads has led to the development and fabrication of adhesive surfaces with potential applications in biomedical materials [121]. For instance, the adhesive strategy of the gecko has been used to develop a hybrid adhesive tape which can be used to guide synthetic adhesives [122]. Another study translated the working mechanism of gecko feet into the development of a biocompatible and biodegradable tissue adhesive for sealing wounds [123]. Similarly, Frost et al. produced a gecko-inspired adhesive based on nanopillars with a diameter in the range of 100–600 nm that effectively bonds to tissue for repair [124]. The sharkskin’s structure can be implemented in engineering designs for drag-reducing and antibiofouling applications [117], as seen in the coating of aeroplanes [125] and ships [126]. Furthermore, the replicated riblet structure can also disrupt the formation of bacterial biofilms, useful for applications on medical devices [127]. Lastly, the unique structural absorption characteristics of feathers seen in Birds of Paradise may have further applications in antireflective materials [128].

### 2.4. Nature-Inspired Architectures to Guide Cell Behaviour

The previous sections highlighted the importance of surface characteristics for a wide variety of applications. However, these properties are also relevant in guiding cell behaviour in vitro, which can be attributed to creating antifouling properties in vivo, for example. Concerning this, natural surfaces have been used in bioinspired approaches to guide cell behaviour, including spider silk [129,130], oyster shells (*Pinctada maxima)* [131,132,133,134,135], lotus leaves [136,137,138,139,140], and cicada and dragonfly wings [36,141,142,143,144,145,146,147].

**Figure 9:**
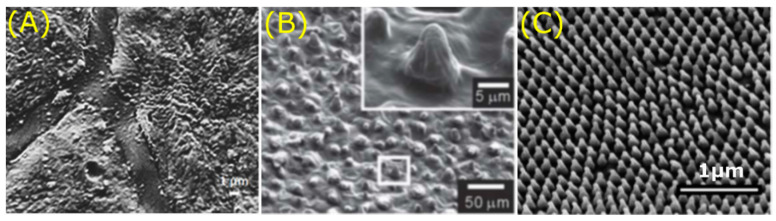
Nature-inspired topographies used to control cell behaviour. (**A**) Replicated prism topography of the oyster shell (*Pinctada maxima)* for phenotypical maintenance of mesenchymal stem cells (MSCs). (**B**) Hierarchical micro- and nanostructures copied from the lotus leaf to increase cell viability. (**C**) Nanotopography imprinted from a cicada wing with bactericidal properties. Scale bars: bottom right. Images adapted with permission from: (**A**) Alakpa et al. [135], The Prismatic Topography of Pinctada maxima Shell Retains Stem Cell Multipotency and Plasticity In Vitro. © (2018), Published by WILEY-VCH Verlag GmbH&Co. KGaA, Weinheim. (**B**) Jeon et al. [137], The effect of microsized roughness in nano/microsized hierarchical surfaces replicated from a lotus leaf on the activities of osteoblast-like cells (MG63). *J. Mater. Chem.* © (2012), The Royal Society of Chemistry; permission conveyed through Copyright Clearance Center, Inc. (**C**) Dickson et al. [36], Nanopatterned polymer surfaces with bactericidal properties. *Biointerphases*, *10*, © (2015), American Vacuum Society.

In the 1910s, Harrison was the first to note the influence of natural substrata on cell behaviour such as cell shape, migration and cytoskeletal organisation [129]. In his experiment, nerve cells of frogs were mounted on spider silk to investigate the response to such solid structures [129]. It was observed that when forced into free hanging drops, cells adapted their shape and became spherical [129]. A few years later, similar observations were made, where cells from the epithelium of the frog showed active movement along the spider web [130]. Nowadays, silk is exploited for bone tissue engineering applications. For example, silk fibroin nanoparticles promote osteogenic differentiation of rabbit adipose-derived stem cells [148]. The osteoinductive properties of shells were already explored several thousand years ago, when Mayans used the shells as tooth replacement [133]. However, not until the early 1990s the potential of nacre, the inner shell layer of molluscs, in stimulating bone formation was observed [131]. Namely, the presence of nacre chips in a culture of human osteoblasts guided the formation of bone nodules [131]. More recently, Green et al. [132] showed the potential of nacre particles and the nacre soluble matrix to induce the early stages of human bone cell differentiation, again showing its osteoinductive capacity. Likewise, the invertebrate shell was used in another study in a similar manner [133]. However, this time the importance of the nacre topography rather than the chemistry in inducing osteogenesis in mesenchymal stem cells (MSCs) was highlighted [133]. Of interest, the prismatic topography (Figure 9A) also allowed maintaining bone-marrow derived MSC phenotype in long-term culture [135] and induced osteogenic differentiation, which was related to an increase in cell spreading [134]. Altogether, the oyster shell provides a promising tool in therapeutic strategies for engineering bone or biomaterial design to maintain multipotent properties.

The lotus leaf has been an inspiration for surface design to control cell behaviour for different purposes [136,137,139,140]. For example, the lotus leaf structure is used to steer cell differentiation [137,139]. In osteoblast like cells (MG63) the hierarchical lotus structures (Figure 9B) induced increased cell viability and calcium deposition compared to flat [137]. Another effect was observed on adipose derived mesenchymal stem cells, where the lotus structure increased adipogenic differentiation, while chondrogenic and osteogenic differentiation were decreased [139]. Furthermore, cell adhesion and proliferation are also modulated by the lotus structure [136,138,140]. The dual micro- and nanostructure influenced cell attachment and proliferation of different cell lines (SaOs-2, L929 and ATDC5), linked to morphological changes [140]. Similarly, the superhydrophobic lotus characteristics prevented adhesion and proliferation of MSCs [136]. These properties were utilised by Mao et al. [138] to fabricate a lotus-like superhydrophobic film with good blood compatibility while no platelets adhered, useful in biomedical devices to prevent coagulation.

Another example of a natural surface able to modulate cell behaviour is the cicada wing, which is able to kill bacteria due to arrays of nanopillars present on its surface, as previously explained [85]. This bactericidal effect has led to the development of cicada inspired nanopatterned surfaces [36,141,142]. For instance, an array of nanopillars with a width of 70 nm, spacing of 100 nm, and a height of 210 nm (Figure 9C) increased bacterial cell death compared to flat and larger nanopillared counterparts [36]. Bacterial cell morphology on these nanopillared surfaces appeared stretched and ruptured, whereas bacteria were rod-shaped on the flat control. In a similar study, the length scale parameters that control spatial patterning of bacteria on a surfaces was investigated [33]. It was concluded that bacterial attachment becomes more disordered as spacing between pillars decreases, with increasing high-aspect-ratio being key in preventing bacterial attachment. In line with this, another study identified that the killing efficiency of nanopillars (*h*: 190 nm, *d*: 80 nm) against *Staphylococcus aureus* bacteria increased by decreasing the interspace between the pillars [141]. Intriguingly, identical disordered nanopillars did not show a similar outcome [141]. Furthermore, multi-directional nanospikes (*d*: 120 nm, *h*; 300 nm, *s*: 200–400 nm) showed biocidal activity against both *Staphylococcus aureus* and *Pseudomonas aeruginosa* bacteria [142].

Potential nanopatterns that can simultaneously direct cell response and kill bacterial cells inspired by insect wings are also described in [143,144,145,146,147]. Such dual biofunctionality was investigated for three nanopatterns with pillar diameters ranging from 122–126 nm, heights between 94–188 nm, and spacing of 300 nm [144]. On these patterns *Escherichia coli* cells were severely damaged and formation of extracellular polymeric substance was disrupted. Similarly, in another study nanopatterns in the shape of pillars with a height of 190 nm, spacing of 170 nm, and a diameter of 80 nm showed significantly higher bactericidal effects compared to nonpatterned surfaces [143]. As nanotopographies in this size range are known to induce osteogenic differentiation in stem cells [149], these topographies can possibly steer cell differentiation and kill bacteria at the same time. Moreover, titania nanowire arrays were able to discriminate bacterial and mammalian cells [145]. While bacteria were eliminated through mechanical rupture, mammalian cell adhesion, and proliferation were guided depending on type of nanoarray [145]. Interestingly, titanium nanoarrays mimicking the dragonfly wings showed a similar response [146]. These surfaces showed selective bactericidal activity, while also enhancing proliferation of primary human fibroblasts [146]. Likewise, similar nanostructured titanium surfaces were able to kill bacteria and enhance the growth of MG63 cells compared to flat controls [147]. Such properties could be relevant for biomedical implants to tackle host–tissue integration problems. Overall, the unique surface characteristics of natural surfaces can be used to regulate cell behaviour useful for different therapeutic and biomaterial applications.

### 2.5. Structure of Extracellular Matrix (ECM) Guides Functional Properties in Human and Animal Tissue

**Figure 10:**
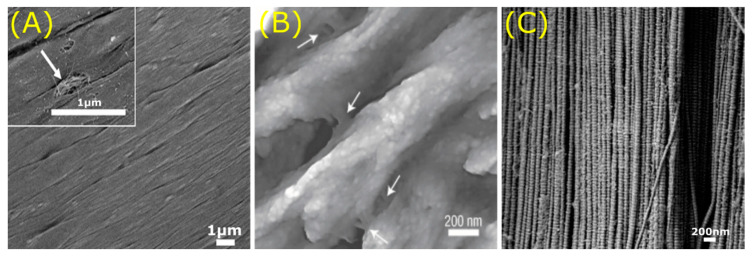
Ensemble of structural organisation found in various types of tissue. (**A**) Top view of ex vivo rat myocardium showing direction of alignment of matrix fibres. (**B**) Structure of individual mineralised collagen fibrils attached to each other by glue filaments (arrows) in bone tissue. (**C**) Collagen fibrils are aligned continuity in mature rat ligaments. Scale bars: bottom right. Images adapted with permission from: (**A**) Kim et al. [154], Nanoscale cues regulate the structure and function of macroscopic cardiac tissue constructs. Proc. Natl. Acad. Sci. © (2010) (**B**) Springer Nature Customer Service Centre GmbH: Springer Nature, Nature Materials, Sacrificial bonds and hidden length dissipate energy as mineralized fibrils separate during bone fracture, Fantner et al., © (2005) [155]. (**C**) Matrix Biol., Provenzano et al. [156], Collagen fibril morphology and organization: Implications for force transmission in ligament and tendon, 71–84, © (2006) Elsevier.

Bodies of multicellular organisms consist of different types of tissue that all have their own distinct role. Tissue, which is widely known as a group of similar cells with a specific function, obtains a great part of its function from the ECM composition [150]. Namely, the distribution of functional and structural molecules such as collagen in the ECM gives each tissue its distinct properties [151]. Moreover, the complex structural organisation among tissues shows a great variety in ECM architecture [152,153]. For example, the muscular tissue of the heart, also known as the myocardium, shows a directional alignment of ECM fibres (Figure 10A) [154]. Consequently, cells are oriented along the anisotropic parallel arrays of the myocardial tissue due to nanotopographical cues found on the ECM. Nanopatterned substrata with similar structural alignment can be used as a scaffold for the construction of implantable engineered cardiac tissue, as shown for polyethylene glycol hydrogels [154]. Altogether, heart tissue acquires its electrophysiological and mechanical functional properties necessary for precise control of cardiac function from the ECM topography.

Similarly, bone tissue also gains its exceptional mechanical properties from the composition and structure of its matrix [155,157]. Interactions between collagen fibrils and non-fibrous organic matrix is facilitated by the nanoscopic arrangement of the bone. The collagen fibrils are interconnected by glue filaments (Figure 10B), which prevent the separation of the bone structure when force is applied [155]. The glue promotes an energy dissipation mechanism by stretching its sacrificial bonds. Structure of the ECM also plays an important role in endothelial vascular membranes found in vascular tissue of the *rhesus macaque* [158]. Topographical features of vascular basement membranes in the blood vessels are composed of a complex meshwork of pores and fibres in the nano- and submicron range (*d*: 1–1000 nm), as seen in the basement membrane architecture of the aorta. These structural properties guide the normal homeostatic state of vascular tissue by controlling endothelial cell behaviour including adhesion, differentiation, and proliferation. Finally, mechanical behaviour in connective tissue such as tendons and ligaments is also determined by the organisation of the ECM [156]. The structural element collagen-I makes up the continuous fibril morphology of this type of tissue (Figure 10C). Force within connective tissue is transferred through these collagen fibres, giving the tissue its mechanical properties. Finally, surface topography of the ECM has also been used in biomimetic tissue engineering approaches of native skin [159], tendon [160], and liver tissue [160]. In short, a big diversity in structure can be identified between various types of tissue such as heart, bone, vascular and connective tissue. The structural organisation of the ECM within a tissue is a key factor in establishing the tissue’s functional effect, which can be utilised in bioinspired tissue engineering methods.

### 2.6. Replication of Native Tissue to Direct Cell Behaviour

**Figure 11:**
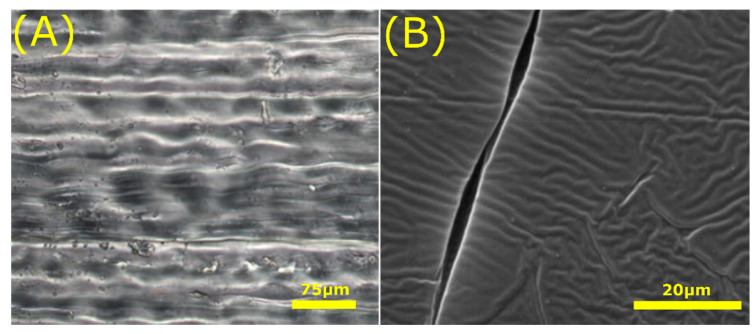
Replicated native tissue structures. (**A**) Replicated elongated and aligned morphology of tendon tissue able to support tenocytic differentiation of MSCs. (**B**) Schwann cell imprinted patterns to direct differentiation of MSCs into Schwann cells. Scale bars: bottom right. Images adapted with permission from: (**A**) Tong et al. [162], Functional replication of the tendon tissue microenvironment by a bioimprinted substrate and the support of tenocytic differentiation of mesenchymal stem cells. *Biomaterials, 33,* © (2012) Elsevier. (**B**) Moghaddam et al. [165], Engineered substrates with imprinted cell-like topographies induce direct differentiation of adipose-derived mesenchymal stem cells into Schwann cells. *Artif. Cells, Nanomedicine, Biotechnol.*, 47, © (2019) Published by Informa UK Limited, trading as Taylor and Francis Group.

A few studies have focused on replicating native tissue to direct cell behaviour [161,162,163,164]. For example, it was shown that by replicating the tendon micro-environment (Figure 11A), mesenchymal stem cells (MSCs) can be guided to differentiate towards a tenogenic phenotype [162]. In another study, the cell shape of mature and de-differentiated chondrocytes were imprinted in polydimethylsiloxane (PDMS), resulting in negative imprinted patterns of these cell surfaces [161]. These patterns directed cellular morphology and expression of chondrogenic markers (collagen-II and aggrecan) in rabbit adipose derived MSCs, depending on the maturation of the chondrocytes used for imprinting.

Using similar approaches, substrates with imprinted osteoblast or Schwann cell topography (Figure 11B) were capable of guiding differentiation of adipose derived MSCs towards the osteogenic [166] and Schwann cell [165] lineage respectively. Lee et al. [163] used UV-assisted capillary force lithography to fabricate a substrate with imprinted nanoscale topography of differentiated skeletal myoblasts. Again, hMSCs cultured on these patterns underwent more efficient commitment to the myogenic lineage compared to the flat control [163]. The relevance of cell shape within tissues was also highlighted by Ron and colleagues [164] using 3D biomimetic engineered biochips and computational models [164]. In the study, human podocytes cultured on the biochips attained a similar shape as seen in vivo. It was concluded that cell shape contains essential information to maintain the cell’s physiologically relevant phenotype, which are dependent on the geometrical constraints imposed upon cells by the surrounding tissue. A consequence can be that the surface to volume ratio of a cell affects reaction and diffusion rates [164]. This changes the expression and subcellular localisation of proteins necessary to manage its function. Furthermore, cell geometry is linked to YAP/TAZ [167] and RhoA [168] signalling, both essential in controlling multiple aspects of cell behaviour such as growth, differentiation and cell cycle maintenance [169,170]. Thus, the shape a cell attains within tissue influences its behaviour. Altogether, replication of native tissue shows high potential for regenerative medicine to guide cell behaviour.

## 3. Discussion

Technological advances in regenerative medicine and tissue engineering rely on the development of functional biomaterials for engineering the cell microenvironment to regulate cell behaviour. Concerning this, a major challenge remains in the design of the right material properties to generate a specific cell response. With the emergence of micro- and nanofabrication techniques and high-content imaging, novel combinatorial and high-throughput approaches have been developed [171,172,173,174]. These libraries are based on miniaturised platforms, which are able to simultaneously characterise a high number of varying surface properties, such as topography [37,38,39,41,43,174] and chemistry [42,174,175,176]. Together with machine learning algorithms this offers a great tool to screen for properties that induce desired cell behaviour in vitro [177]. For example, the TopoChip [37], BSSA [38], and MARC [39] platforms have investigated the relationship between topography and cell response. Additionally, in the field of material science these automatic measurement methods are also used to screen for functional properties, ranging from structural to optical characterisations [41,42,43]. However, these high-throughput platforms also have limitations, since they only vary a limited number of parameters and therefore each focus on a restricted area of the biomaterial design space. Cell studies on artificial surfaces have proven that topographical cues are of great importance in controlling cell behaviour. For example, cell shape is highly influenced by surface topography, which can influence several cellular processes ranging from migration to differentiation [164,167,178]. In vivo, it is known that cells respond to the dual-scale structures of the ECM both at the micro- and nanoscale. Interestingly, natural surfaces possess properties that are known to influence cell behaviour both in vitro and in vivo. Namely, natural surfaces show hierarchical structures, a high degree of surface roughness and a large diversity of patterns, steering wettability in all regimes as seen in plants, insects, and animals. Thus, natural surfaces can be utilised in bioinspired design because of their unique surface properties, not found on artificial surfaces used in conventional cell studies [63]. This method benefits from its focused approach by using natural surface properties to regulate cell behaviour without the need for intensive screening or in silico design. Such development of novel biomaterials can also be applied to three-dimensional microenvironments, ranging from apple-derived cellulose scaffolds [179] to biomimetic marine sponge fibre skeletons for tissue regeneration [180]. Biomimetic research in this area has also turned towards the replication of native tissue and cell structures to modulate cell behaviour or even to harnessing the potential of decellularized ECMs as a three-dimensional natural architecture for cell support and growth [181,182]. Further translation of natural architectures into the field of regenerative medicine and tissue engineering opens up opportunities in the clinic. Integration of their (multi)functional properties can aid to reduce implant associated infections, increase the biocompatibility of medical devices, and incorporate controlled release systems in scaffolds. Advances in these domains will enhance biomaterials in their ability to function in intimate contact with living tissues.

## 4. Conclusions

The utilisation of natural surfaces as templates for fabrication of artificial surfaces for cell studies can bring about novel cell responses and unravel the mechanisms involved in the interplay between material characteristics and cell phenotype. Together with high-throughput and machine learning methods this can provide a solution to find optimal surface parameters for regulating cell behaviour. In conclusion, biomimicry of natural surfaces has a great potential to enhance technologies in the field of regenerative medicine and tissue engineering through advances in the ability of functional biomaterials to guide cell behaviour.
